# Case report: *NUDT15* polymorphism and severe azathioprine-induced myelosuppression in a young Chinese female with systematic lupus erythematosus: a case analysis and literature review

**DOI:** 10.3389/fphar.2023.1001559

**Published:** 2023-05-09

**Authors:** Juan Gu, Yupei Lin, Yuhe Wang

**Affiliations:** ^1^ Department of Pharmacy, Affiliated Hospital of Zunyi Medical University, Zunyi, China; ^2^ Department of Rheumatology and Immunology, Affiliated Hospital of Zunyi Medical University, Zunyi, China

**Keywords:** azathioprine, myelosuppression, the nucleoside diphosphate-linked moiety X motif 15, *NUDT15*, gene polymorphism

## Abstract

Azathioprine is clinically used as an immunosuppressant for treating autoimmune diseases. However it has narrow therapeutic indices due to frequent myelosuppression. Polymorphic variants of genes coding for thiopurine S-methyltransferase (*TPMT*) and nucleoside diphosphate-linked moiety X motif 15 (*NUDT15*) are critical determinants of AZA intolerance, and the differences in frequencies of the two genetic variants exist among people of different ethnicities. Most reports regarding *NUDT15* variant, AZA-induced myelosuppression occurred in patients with inflammatory bowel disease and acute lymphoblastic leukemia. Moreover, detailed clinical characteristics were not frequently reported. Here we present the case of a young Chinese female with the *NUDT15* c.415C>T (rs116855232, TT) homozygous variant and wild-type TPMT*2 (rs1800462), TPMT*3B (rs1800460), and TPMT*3C (rs1142345) who received high doses of AZA (2.3 mg/kg/d) for systematic lupus erythematosus and had not been told to undergo routine blood cell counts during AZA ingestion. The patient had suffered from severe AZA-induced myelosuppression and alopecia. Moreover, dynamic changes in blood cell counts and responses to treatment were observed. We also conducted a systematic review of published case reports of patients exclusively with *NUDT15* c.415C>T homozygous or heterozygous variants to review the characteristics of dynamic changes in blood cells so as to provide reference information for clinical treatment.

## 1 Introduction

Azathioprine (AZA) is a prodrug for mercaptopurine (6-MP) that exhibits similar pharmacologic effects to those of other thiopurines (6-MP and thioguanine) and is clinically used as an immunosuppressant for treating severe rheumatoid arthritis (RA), systemic lupus erythematosus (SLE), dermatomyositis, autoimmune chronic active hepatitis, and spontaneous thrombocytopenic purpura. Thiopurines have narrow therapeutic indices owing to frequent myelosuppression commonly characterized by neutropenia and sometimes associated with thrombocytopenia and aplastic anemia (Montgomery et al., 2022).

Upon administration, AZA is first nonenzymatically converted into 6-MP and then enzymatically metabolized into pharmacologically active 6-thioguanine nucleotides (6-TGN) and deoxythioguanosine phosphates. Thiopurine S-methyltransferase (TPMT), which strongly inactivates thiopurine metabolites (Booth et al., 2011), and nucleoside diphosphate-linked moiety X motif 15 (NUDT15), which dephosphorylates thiopurine effector metabolites such as TdGTP and inhibits active metabolite loading onto nucleic acids (Yang et al., 2014), are two major enzymes that work alongside other factors, such as intestinal microbial enzymes (Wang et al., 2022), to influence AZA metabolism. *TPMT* genetic polymorphisms and *NUDT15* variations are therefore critical determinants of thiopurine intolerance (Cargnin et al., 2018). *TPMT* single nucleotide polymorphisms (SNPs) result in unstable proteins and enhance TPMT degradation (Relling et al., 2019). Variants TPMT*2, TPMT*3A, TPMT*3B, and TPMT*3C are relatively common in Caucasians and Africans, and TPMT*3C is the most frequent variant in Asians, although its frequency is still lower than that of European populations (Kishibe et al., 2018; Su et al., 2020; Chen et al., 2021). *NUDT15* is known to have 26 alleles, and NUDT15*2 (p.V18_V19insGV and c.415C>T), *3 (c.415C>T), and *9 (c.50delGAGTCG) are recognized as loss-of-function variants (Tanaka and Saito, 2021). *NUDT15* c.415C>T influences protein stability, inhibits or prevents enzyme activity (Valerie et al., 2016), and is relatively common in East Asians and Hispanics but less frequent in Europeans (Moriyama et al., 2016). In Chinese populations, the heterozygote and homozygote frequencies of *NUDT15* c.415C>T are 19.95% and 1.97%, respectively (Chen et al., 2021). A report showed that the incidence of severe myelosuppression in patients with homozygote *NUDT15* c.415C>T taking 0.85 (0.5–1.09) mg/kg/d AZA was 85.7% (Yang et al., 2014).

Herein, we present a case study of a young Chinese female with SLE taking 2.3 mg/kg/d AZA and experiencing severe AZA-induced myelosuppression and alopecia due to a failure to perform AZA metabolism-related genotyping prior to treatment initiation and a lack of routine blood cell counts during AZA administration. The genotype of the patient was revealed to include the *NUDT15* c.415C>T(TT) homozygous variant with wild-type TPMT*2, TPMT*3B, and TPMT*3C, as detected when myelosuppression appeared, and these characteristics are described to provide a reference for clinical treatment not only to assess status changes of myelosuppression when it happened but also to remind physicians to perform genotyping before prescribing AZA to determine whether AZA is suitable for patients. Moreover, we present the results of a systematic review of published case reports in order to characterize dynamic changes regarding the severity of AZA-induced leukopenia in patients with *NUDT15* c.415C>T homozygous or heterozygous variants alone with the purpose to provide more reference information for clinical treatment of AZA-induced myelosuppression in specific patients with only *NUDT15* c.415C>T variants.

## 2 Case presentation

**FIGURE 1:**
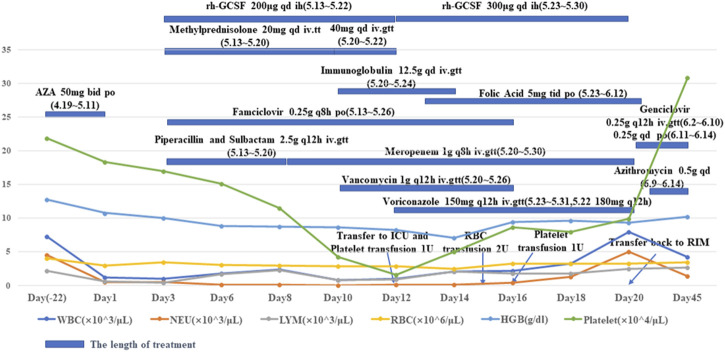
White blood cell, hemoglobin, and platelet counts for a young Chinese patient with SLE and schedule of treatment. WBC: white blood cells; NEU: neutrophils; LYM: lymphocytes; RBC: red blood cells; HGB: hemoglobin; RIM: rheumatic immunology department. AZA: azathioprine tablet; Rh-GCSF: recombinant human granulocyte colony stimulating factor injection; Methylprednisolone: methylprednisolone sodium succinate for injection; Immunoglobulin: human immunoglobulin for intravenous injection; Folic: folic acid tablets; Piperacillin and sulbactam: piperacillin sodium and sulbactam sodium for injection; Meropenem: meropenem for injection; Azithromycin: azithromycin for injection; Vancomycin: vancomycin for injection; Voriconazole: voriconazole for injection; Famciclovir: famciclovir dispersible tablets.

An 18-year-old female Chinese patient was diagnosed with SLE at the age of 15 years according to the 2012 Systemic Lupus International Collaborating Clinics (SLICC) criteria. The patient presented with clinical manifestations of oral ulcers, cutaneous lupus, nephritis, and both antinuclear (ANA) and antiphospholipid antibodies on 19 August 2018. After 3 years of treatment with hydroxychloroquine (200 mg BID) and prednisone (starting with 30 mg QD with gradual reduction), hydroxychloroquine was discontinued. During the prednisone dose reduction, the attending doctor prescribed her AZA (50 mg bid, 2.32 mg/kg/d) on 19 April 2022, without identifying the presence of AZA metabolism-related genes, likely due to negligence of the physician or lack of knowledge regarding AZA metabolism-related gene testing. On 11 May 2022 (after taking AZA for 22 d), this patient was admitted to a local hospital with a fever (axillary temperature of 38.9 °C), pharyngalgia accompanied by pigmentation of fingertip joint skins, and alopecia. Routine blood tests revealed a white blood cell count of 0.45 × 10^9^ cells/L with normal liver and kidney function. The physician considered the above symptoms adverse reactions to AZA and suggested stopping AZA administration and transferring the patient to the Rheumatic Immunology Department of our hospital. This patient was admitted to our hospital on 13 May 2022 (day 3 after withdrawing AZA). A blood sample was collected to assess AZA metabolism-related genes *TPMT* and *NUDT15* using commercially outsourced next-generation sequencing (Guizhou KingMed Center for Clinical Laboratory, Guiyang city, China) (Table 1) and to determine blood cell counts (Table 1 and Supplementary 1) and liver and kidney function. Liver and kidney function were normal, and antibody testing results were weakly positive for ANAs and negative for antiphospholipid antibodies. No cutaneous lupus was found, and SLE activity was considered stable. The patient exhibited rapid onset of alopecia, and no hair was observed approximately 10 d after discontinuing AZA treatment. During discontinuation, the patient was given folic acid (10 mg TID p.o.) to compete with AZA for hypoxanthine binding; recombinant human granulocyte colony-stimulating factor (150 μg QD∼300 μg BID i.h.); recombinant human thrombopoietin injection (15,000 U QD i.h.) to increase leucocyte and platelet counts; methylprednisolone to reduce inflammation; and meropenem, vancomycin, voriconzole, and famciclovir to treat infections (Figure 1). Despite treatment to promote leukocyte and platelet production, the lowest platelet count of 15 × 10^9^ platelets/L was observed on day 11 (with multiple subcutaneous ecchymosis and negative platelet antibody results), 2 d after the lowest white blood cell count. Platelet recovery began 2 d after that of white blood cells. During treatment, the patient was transferred to the Intensive Care Unit (ICU) with a neutrophil count of 0.00 × 10^9^ cells/L and received supportive care for 10 d. Neutrophil count remained below 0.05 × 10^9^ cells/L for 9 d before the patient was transferred back to the Rheumatic Immunology Department. The patient recovered from myelosuppression gradually, and white blood cell counts were recovered on day 18 after AZA discontinuation (Supplementary 1). The patient was discharged from hospital on 15 June 2022 (day 46), and attending physicians chose hydroxychloroquine (200 mg BID) as the follow-up treatment. The total cost of hospitalization was 83,749.00 Chinese yuan, based on the hospital information system. Informed consent was obtained from the patient for publication of the case report.

**TABLE 1:** Reported characteristics of patients with homozygous variant *NUDT15* c.415C>T genotypes taking azathioprine (AZA).

No.	Source	Age/Gender	Disease	*TPMT* genotype	*NUDT15* c.415C>T genotype	AZA dose	Interval^a^ and neutrophil counts (× 10^9^ cells/L)	The lowest NEU^b^ counts (× 10^9^ cells/L)	Recovery period (NEU count above 0.5×10^9^ cells/L or 0.1×10^9^ cells/L) after discontinuation^c^ (other details)
		Ethnicity							
		Weight							
1	This report	18 Female	SLE^d^	*2, c.238G>C(GG); *3B, c.460G>A(GG); *3C, c.719A>G(AA)	TT	2.32 mg/kg/d	27 d 0.43	0.00	12 d, 7 d (Neutrophil count 2.42 × 10^9^ cells/L at day 18 after discontinuation)
		Chinese							
		43 kg							
2	Séverine et al. (2022)	26 Female	CD^e^	*2, c.238G>C(GG)	TT	2.5 mg/kg/d	35–42 d 0.66	∼0.3	? (Neutrophil count 1.5 × 10^9^ cells/L at day 4 after discontinuation ^(^
		European		*3B, c.460G>A(GG)					
		?		*3C, c.719A>G(AA)					
3	Bae et al. (2020)	12 Female	CD	TPMT *1/*1	TT	0.5 mg/kg/d	∼20 d 3.37 (due to significant hair loss)	?	?
		Korean							
		34 kg							
4	Yan et al. (2018)	47 Male	PV^f^	TPMT *1/*1	CT	150 mg/d	65 d ?	?	?
		Chinese							
		?							
5	Shih et al. (2019)	28 Female	PV	TPMT *1/*1	TT	100 mg/d	28 d 0.36	0.36	?
		Chinese							
		?							
		40 Female	PV	TPMT *1/*1	TT	100 mg/d	28 d 0.3	0.3	?
		Chinese							
		?							
6	Nomura et al. (2018)	61 Female	PV	*?*	TT	100 mg/d	14 d ?	0.435	8 d
		Japanese							
		?							
		57 Female	PV	*?*	TT	100 mg/d	17 d ?	0.21	10 d
		Japanese							
		?							
		37 Female	RA^g^	*?*	TT	50 mg/d twice a week	46 d ?	1.34	5 d
		Japanese							
		?							
7	Hon et al. (2018)	17 Female	Eczema	TPMT *1/*1	TT	50 mg/d	28 d 0.8	0.1	? (Neutrophil count 1.8 × 10^9^ cells/L at day 7 after discontinuation)
		Chinese							
		70 kg							
8	Devasia et al. (2020)	26 Female	AIHA^h^	*2, c.238G>C(GG)	TT	1.5 mg/kg/d	7 d 0.19	?	? (Presented with hair loss, oral ulcers, and leukopenia)
		Indian		*3B, c.460G>A(GG)					
		?		*3C, c.719A>G(AA)					
		28 Female	Chronic ITP^i^	*2, c.238G>C(GG)	TT	1.5 mg/kg/d	14 d 0.21	?	? (Presented with hair loss and leukopenia)
		Indian		*3B, c.460G>A(GG)					
		?		*3C, c.719A>G(AA)					
		34 Female	Chronic ITP	*2, c.238G>C(GG)	TT	2 mg/kg/d	14 d 0.19	?	? (Presented with fever and cytopenia)
		Indian		*3B, c.460G>A(GG)					
		?		*3C, c.719A>G(AA)					
		18 Female	Chronic ITP	*2, c.238G>C(GG)	CT	1.5 mg/kg/d	21 d 1.68	?	? (Presented with hair loss, blood counts were normal, reduced doses of 1 mg/kg/d were tolerated)
		Indian		*3B, c.460G>A(GG)					
		?		*3C, c.719A>G(AA)					
		12 Female	Chronic ITP	*2, c.238G>C(GG)	CT	2 mg/kg/d	28 d 0.8	?	? (Presented with hair loss and leukopenia, lost to follow-up)
		Indian		*3B, c.460G>A(GG)					
		?		*3C, c.719A>G(AA)					
9	Fei et al. (2018)	22 Female	SS^j^	*3C, c.719A>G(AA)	TT	0.91 mg/kg/d	21 d 0.5	0.1	5 d, 3 d (Neutrophil count of 2.0 × 10^9^/L at day 10 after discontinuation)
		Chinese							
		55 kg							
10	Ben Salem et al. (2020)	42 Male	IBD^k^	?	TT	2.27 mg/kg/d	21 d 0.64 (White blood cell count)	?	(White blood cell count of 5.5 × 10^9^/L at day 10 after discontinuation)
		Tunisian							
		?							

^a^ Interval: from taking AZA to discovery of myelosuppression.

^b^ NEU: Neutrophils.

^c^ NEU count above 0.5×10^9^/L or 0.1×10^9^/L.

^d^ SLE: Systematic lupus erythematosus.

^e^ CD: Crohn’s disease.

^f^ PV: Pemphigus vulgaris.

^g^ RA: Rheumatoid arthritis.

^h^ AIHA: autoimmune hemolytic anemia.

^i^ ITP: Idiopathic thrombocytopenic purpura.

^j^ SS: Sjögren syndrome.

^k^ IBD: inflammatory bowel disease; ?: not mentioned. Case reports of patients who took other medicine interacting with AZA, such as allopurinol (Takeuchi et al., 2021) or patients with abnormal liver function (Ailing et al., 2016) are not listed in this table. Other vaiants of *NUDT15*, such as *NUDT15* 416G>A (Tomiyoshi et al., 2020), are also not listed.

Three months after patient discharge, follow-up revealed that the patient followed medical advice to repeat routine blood tests at the end of the third week after discharge. Results indicated that white blood cell counts had effectively recovered, and the immunosuppressant tacrolimus was added as an SLE treatment. Two months and 2 weeks after discharge, routine blood tests showed a normal range for white blood cell count (4.86 × 10^9^ cells/L), with a neutrophil proportion of 34%, a little lower than normal. Her hair had grown to 3.5 cm and was sparse but without other discomfort. Notably, because of her hospitalization, she missed a college entrance examination and had to repeat her studies for another year before taking the exam. Her mother quit her job to take care of her, which had an impact on their income. When we reached out to her mother, she complained that the doctor failed to recommend a genotype test before prescribing AZA to her daughter and wished for compensation from the hospital.

## 3 Literature review

We conducted this review with the goal of providing specific information about neutrophil counts and AZA administration and recovery durations in patients with *NUDT15* c.415 C>T only. Therefore, articles describing patients with variants of other sites of *NUDT15* or other genes were excluded. In addition, case reports of patients who took medicines interacting with AZA or patients with abnormal liver function are not included in order to reduce the impact of other factors on AZA metabolism. We searched PubMed with retrieval terms “((((“Leukopenia”[Mesh]) OR (((Leukopenias) OR (Leukocytopenia)) OR (Leukocytopenias))) OR (myelosuppression)) AND (((“Azathioprine”[Mesh]) OR (azathioprine)) OR (((Imuran) OR (Immuran)) OR (Imurel)))) AND (NUDT15)” for article published from inception to 31 July 2022 and then performed a secondary search of cited documents for our bibliography retrieval. A total of 67 articles were found. After reading the titles and the full texts, nine reports that included specific neutrophil counts and medication regime-related information were retrieved (Table 1).

## 4 Results and discussion

A study including 70 Chinese patients with SLE or RA demonstrated that the frequency of TPMT was 1% whereas that of NUDT15 was 14%. Among NUDT15 variants, wild-type, heterozygous, and homozygous NUDT15 c.415C>T genotypes were carried by 51 (72.9%), 18 (25.7%), and 1 (1.4%) patients, respectively (Su et al., 2020), and by 86.9%, 11.5%, and 1.5% of 60 Indian patients, respectively (Shah et al., 2017). Although the homozygote frequency of *NUDT15* c.415C>T is lower (1.97%) than that of heterozygote (19.95%) in Chinese populations (Chen et al., 2021), incidence of severe myelosuppression in patients with homozygous *NUDT15* c.415C>T taking 0.85 (0.5–1.09) mg/kg/d AZA was high (85.7%) (Yang et al., 2014). We aimed to report the clinical characteristics and response to treatment of a young Chinese female the *NUDT15* c.415C>T (TT) homozygous variant and SLE suffering from severe AZA-induced myelosuppression due to failure of the attending physician to perform AZA metabolism-related genotyping before administering AZA and to recommend routine blood cell counts analysis during AZA administration. This case description provides a valuable reference for clinical treatment and serves as a reminder to physicians to perform genotyping before prescribing AZA to patients. We also conducted a systematic review of published case reports to characterize dynamic changes regarding the severity of AZA-induced leukopenia in patients with *NUDT15* c.415C>T homozygous or heterozygous variants.

To the best of our knowledge, this is the first report of a patient with SLE and the homozygote *NUDT15* c.415C>T variant who ingested high doses of AZA for 22 d and exhibited severe myelosuppression. The lowest initial neutrophil count was 0.00 × 10^9^ cells/L and neutrophil counts were below 0.05 × 10^9^ cells/L for 9 d during hospitalization, with a recovery period of 18 d after discontinuation of AZA; this is the longest recovery time reported. A report indicated that a European female patient with Crohn’s disease and the homozygous *NUDT15* c.415C>T variant ingested a higher dose of AZA (2.5 mg/kg/d) for 35–42 d before withdrawal. However, the lowest neutrophil count in this patient was 0.3 × 10^9^ cells/L and the recovery period was only 4 d after discontinuation of the drug (Séverine et al., 2022). According to one case report (Shih et al., 2019), patients with the same genotype, disease, and ethnicity taking similar doses for a similar period may develop myelosuppression with similar characteristics. Whether the difference in AZA tolerance between the Chinese and European patients was due to disease or ethnicity differences requires further investigation.

Moriyama *et al.* (Moriyama et al., 2016) reported that multiple heterozygous (c.415C > T; c.416G > A) and homozygous (c.415C > T) *NUDT15* variant proteins exhibit similar enzymatic activity *in vitro*. Otsuka *et al.* (Otsuka et al., 2019) reported a 57-year-old Japanese man with SLE and multiple heterozygous *NUDT15* variants (c.415C > T; c.416G > A) who had ingested a lower dose of AZA (50 mg/d) than the patient in our report and presented with severe AZA-induced leukopenia and neutropenia, which was sustained for 4 weeks (longer than our female Chinese patient). Whether this discrepancy is due to differences in ethnicity or *NUDT15* genetic variants remains to be elucidated.

The case of a young Chinese patient described here serves to remind physicians to perform genotyping before prescribing AZA to determine whether AZA is suitable for patients. Starting doses of AZA should be adjusted based on *TPMT* and *NUDT15* genotypes to reduce the incidence of adverse drug reactions (Relling et al., 2019). Patients with *NUDT15* heterozygous genotypes received 50 mg AZA daily, while homozygotes were recommended to take alternative drugs (Tanaka and Saito, 2021). If there are not alternative drugs available for patients with homozygous genotype of *NUDT15* c.415C > T, we recommend a dose of 0.2 mg/kg/d AZA as the initial dose, as described by Fan *et al.* (Fan et al., 2019). If *NUDT15* genotyping is unavailable, initiation of AZA therapy at a low dose, such as 0.5 mg/kg/day (Yang et al., 2014), is recommended, along with close observation of hair loss and blood cell counts within 2 weeks. This may represent an alternative method for preventing thiopurine-induced early leukopenia in Asian patients. According to Table 1, leukopenia may occur in patients with homozygous *NUDT15* c.415C>T variant genotypes within 7–14 d of AZA administration onset. In Asian populations for whom genotyping is unavailable, we recommend weekly monitoring of white cell counts for at least 1 month to ensure early identification of myelosuppression; further testing should be conducted every 2 weeks and then once a month. Moreover, if it is needed, monitoring of 6-TGN target levels may also be a consideration (Kang et al., 2020).

Based on the findings described above, it is inappropriate to prescribe AZA without first testing for the presence of treatment-related genes and then performing routine monitoring of blood cell counts during AZA administration. The failure to perform these steps led to serious myelosuppression in a patient who was hospitalized for more than 1 month, at a total cost of 83,749.00 Chinese yuan, whereas the cost of genetic testing is only 380.00 Chinese yuan. Furthermore, because of her hospitalization, this patient failed to take the annual college entrance examination that is required for every student, setting her back academically. Her parents expressed the intention to claim compensation, which would cause great economic and reputative losses to the hospital. In order to avoid the recurrence of such an event, as clinical pharmacists, we disseminated the information related to this medication (specifically the use of thiopurines) to physicians in our hospital.

There are some limitations to this study. In this patient, only *NUDT15* c.415C>T; TPMT *2, 238 G>C; TPMT *3B, c.460 G>A; and TPMT *3C, 719 A>G were detected based on their relatively high frequency in China. Full-length *TPMT* and *NUDT15* sequencing was not performed. Moreover, we conducted this review with the goal of providing specific information about neutrophil counts and AZA administration and recovery durations in patients with *NUDT15* c.415 C>T only. Therefore, articles describing patients with variants of other sites of *NUDT15* or other genes were excluded. Although we conducted a review to identify dynamic changes in myelosuppression induced by AZA in patients with *NUDT15* c.415C>T, such dynamic changes in myelosuppression were rarely described. Due to the low frequency of homozygous variant *NUDT15* c.415C>T genotype and incidence of severe myelosuppression in patients with homozygous variant *NUDT15* c.415C>T genotype, further detailed case reports are required to illustrate the clinical characteristics of patients taking AZA exclusively with the homozygous variant *NUDT15* c.415C>T genotype. We believe that our study makes a significant contribution to the literature because it provides references for physicians to treat patients with homozygous variant *NUDT15* c.415C>T genotype and suffered from AZA-induced severe myelosuppression. More importantly, our report will remind clinicians of the importance to test the metabolism-related genes prior to prescribing AZA as well as routine blood during patients taking AZA.

## Data Availability

The datasets for this article are not publicly available due to concerns regarding participant/patient anonymity. Requests to access the datasets should be directed to the corresponding authors.
